# A phage protein-derived antipathogenic peptide that targets type IV pilus assembly

**DOI:** 10.1080/21505594.2021.1926411

**Published:** 2021-05-19

**Authors:** In-Young Chung, Bi-o Kim, Ju-Hyun Han, Jonggwan Park, Hee Kyoung Kang, Yoonkyung Park, You-Hee Cho

**Affiliations:** a Department of Pharmacy, College of Pharmacy and Institute of Pharmaceutical Sciences, CHA University, Seongnam-si, Korea; b Department of Biomedical Science, Chosun University, Gwangju, Korea

**Keywords:** *Pseudomonas aeruginosa*, phage, antipathogenic, peptide, motility, PilB

## Abstract

Phage-inspired antibacterial discovery is a new approach that recruits phages in search for antibacterials with new molecular targets, in that phages are the biological entities well adapted to hijack host bacterial physiology in favor of their own thrive. We previously observed that phage-mediated twitching motility inhibition was effective to control the acute infections caused by *Pseudomonas aeruginosa* and that the motility inhibition was attributed to the delocalization of PilB, the type IV pilus (TFP) assembly ATPase by binding of the 136-amino acid (aa) phage protein, Tip. Here, we created a series of truncated and point-mutant Tip proteins to identify the critical residues in the Tip bioactivity: N-terminal 80-aa residues were dispensable for the Tip activity; we identified that Asp82, Leu84, and Arg85 are crucial in the Tip function. Furthermore, a synthetic 15-aa peptide (P1) that corresponds to Leu73 to Ala87 is shown to suffice for PilB delocalization, twitching inhibition, and virulence attenuation upon exogenous administration. The transgenic flies expressing the 15-aa peptide were resistant to *P. aeruginosa* infections as well. Taken together, this proof-of-concept study reveals a new antipathogenic peptide hit targeting bacterial motility and provides an insight into antibacterial discovery targeting TFP assembly.

## Introduction

The rapid emergence and dissemination of antimicrobial resistance (AMR) poses a serious threat to global public health [1]. With the advent of bacterial pathogens that acquired resistance to multiple classes of antimicrobials, not just one, it has become extremely challenging to control infectious diseases. Despite multidimensional worldwide efforts to address the AMR and multi-drug resistance (MDR), the most fundamental solution for this crisis is considered to discover and develop novel antimicrobial drugs in a timely manner.

Due to the shortage of conventional antibiotic pipelines, alternative antibacterial strategies are urgently needed [2,3] and the therapeutic use of phages and the phage-inspired antibacterial discovery have attracted much attention as promising alternative approaches to tackle the AMR and MDR problems [4,5]. Besides the conventional phage therapy, combination of phages and antibiotics, exploitation of engineered synthetic phages and phage-derived enzymes have been considered to treat bacterial infections [6,7]. Recent advances in our understanding of the phage lifecycles have further revealed that phages possess an arsenal of strategies to reprogram the host cellular physiology, in addition to killing the host bacteria [8,9]. In most cases, phage-derived proteins disrupt the key host processes involved in transcription, translation, metabolism, cell division, motility and CRISPR-mediated immunity by interfering with their targets [10–13]. These traits of phages led us to identify phage protein(s) to specifically target the most vulnerable host proteins involved in bacterial growth, virulence and/or survival and to develop phage-inspired antibacterials with new antibacterial mechanisms.

We previously demonstrated that *Pseudomonas aeruginosa* temperate phage D3112 inhibits type IV pilus (TFP)-mediated twitching motility of its host bacterium [14]. Twitching motility is a bacterial group behavior for migration on a surface or spreading into host tissues by extension and retraction of TFPs [15,16]. Besides twitching motility, the TFPs are required for initial attachment and biofilm formation, which are deemed required for the full virulence [17,18]. Thus, the function of TFPs might be considered as an antipathogenic target. The chemical inhibitors for pili or fimbriae formation are called “pilicides”, as exemplified by bicyclic 2-pyridones that inhibits P pilus assembly by sequestering the pilus chaperon PapD in uropathogenic *Escherichia coli* [19,20]. We have evaluated the antibacterial efficacy of D3112 using murine and *Drosophila* infection models [14]: treatment of D3112 significantly reduced mortality and bacterial loads in both *P. aeruginosa*-infected mouse and *Drosophila* animals by inhibiting TFP function, rather than by killing the infected bacteria. Subsequently, we identified a phage protein, Tip that inhibits twitching motility of *P. aeruginosa* [21]: Tip directly binds to its bacterial target PilB, the TFP extension ATPase of *P. aeruginosa* and was sufficient to interfere with the assembly of TFP appendage by antagonizing PilB.

Based on the involvement of TFP-mediated twitching motility in acute virulence and biofilm formation of *P. aeruginosa*, we began to explore the potential of Tip as an antipathogenic platform that targets the bacterial motility. In this study, we identified the critical region (56-aa) at the C-terminal half and some residues (D82, L84, and R85) for the Tip bioactivity by truncation and point mutation analyses. We designed 6 Tip-derived peptides (P1~ P6) based on the critical 56-aa region of Tip. Among the synthetic peptides, P1 from Leu73 to Ala87, which was sufficient to inhibit the twitching motility and sequester for PilB upon exogenous administration. These results suggest that P1 could be an antipathogenic peptide that is inspired by phage lifecycle to develop antibacterials with new antibacterial mechanisms.

## Results

### The C-terminal 56 amino acids of Tip are sufficient for twitching inhibition

**Figure 1.**
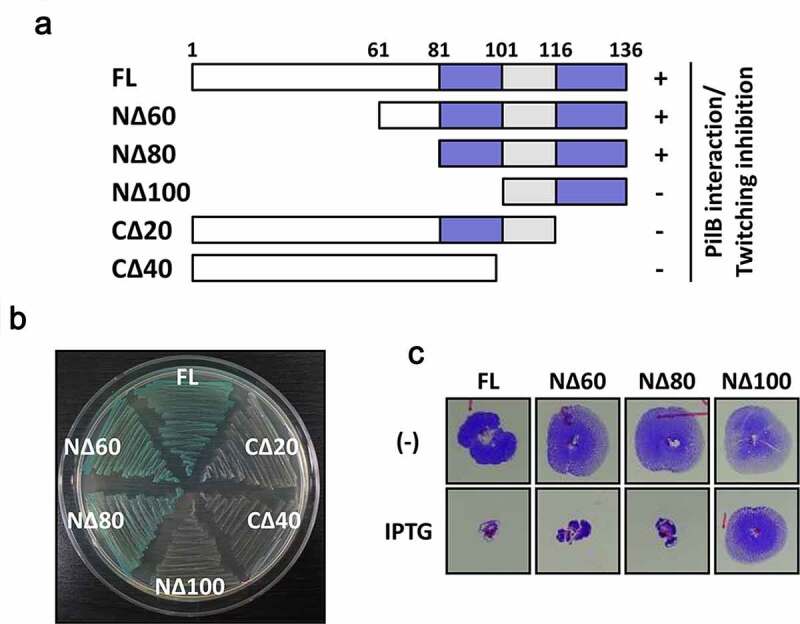
Identification of the region of Tip critical in PilB inhibition

Tip is a 136-aa protein that has no sequence similarity to the proteins with defined functions. It contains a serine-rich region at the N-terminal half and potential helices at the C-terminal region, presumably for macromolecular interactions. To determine the interaction domain of Tip for its bacterial target PilB, we created a series of the truncated Tip mutants (NΔ60, NΔ80, NΔ100, CΔ20 and CΔ40) (Figure 1(a)). The interaction between the truncated mutants and PilB were assessed by bacterial two-hybrid assay as described previously [21]. Except for NΔ60 and NΔ80, all of the truncated Tip mutants were defective in PilB interaction (Figure 1(b)), suggesting the N-terminal 80 aa residues are dispensable for PilB interaction. In contrast to the NΔ80 mutant, the NΔ100 mutant exhibited a complete loss of twitching inhibition (Figure 1(c)), indicating that the 20-aa region (Leu81 to Ser100) is required for the interaction with PilB. Interestingly, the truncation of the 20-aa from the C-terminal end resulted in the loss of PilB interaction, suggesting that the 20 C-terminal amino acids are also critical in PilB interaction. It is clearly demonstrated that the N-terminal 80-aa region of Tip is not essential and that the 20-aa region from Leu81 to Ser100 and the C-terminal region are critical in PilB inhibition.

(a) Schematic representation of the truncated Tip derivatives and their activities. The deletions at the N-terminus (NΔ60, NΔ80, and NΔ100) and at the C-terminus (CΔ20 and CΔ40) from Tip were constructed to determine the regions critical for the Tip activity. They are either deficient (-) or proficient (+) in the Tip activity (PilB interaction and twitching inhibition) as shown in b and c. The critical regions for the Tip activity are designated in blue colors with the amino acid numbers. (b) Bacterial two-hybrid assay with the truncated Tip derivatives was performed to determine the PilB-binding region. The full-length (FL) Tip and the truncated derivatives in A were cloned into pKT25 and then introduced to *E. coli* DHP1 containing pUT18C-PilB. The cells with Tip-PilB interaction were examined by β-galactosidase activity. (c) Twitching motility was assessed using the PA14 cells harboring the full-length (FL) Tip and the N-terminally truncated Tip derivatives in a, which were introduced at the *att*Tn*7* site as described elsewhere [21]. Twitching motility was examined in the absence (-) or presence of 1 mM IPTG.

### A synthetic peptide (P1) inhibits the PilB function upon exogenous administration

**Figure 2.**
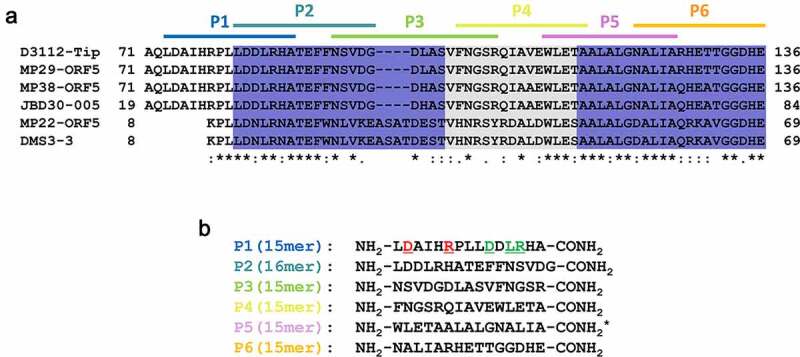
Design and synthesis of a series of Tip-derived peptides

**Figure 3.**
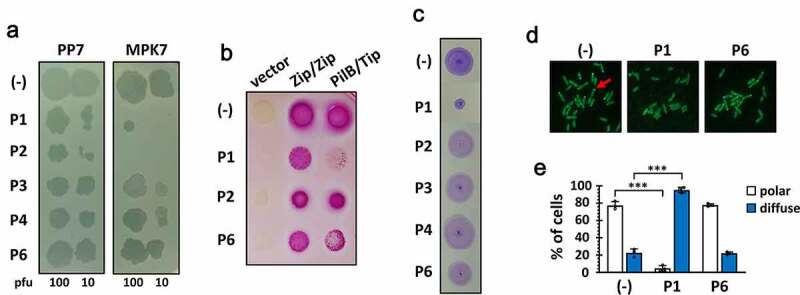
Bioactivity of the Tip-derived peptides upon exogenous administration

A series of 6 peptides (P1~ P6) comprising the C-terminal 56-aa region of Tip were designed. The peptides were chemically synthesized as 15–16 mers with 5–6 aa overlaps (Figure 2(a)) to span the 56-aa region sufficient for the Tip function (Figure 1). The requirement of the bipartite regions (dark blue in Figure 2(a)) for the Tip function led us to an assumption that one region might be preponderantly required for the Tip-PilB interaction and the other might support the interaction. To improve the peptide stability, we introduced amidation to the peptides at the C-termini [22]. Then we examined whether or not at least one of the peptides could represent the Tip activity upon exogenous administration. P5 was excluded due to its poor aqueous solubility, but P2 was included despite its viscosity upon solubilization. We tested them for the capability of PilB dysfunction, which could be assessed by four different readouts (Figure 3). As shown in Figure 3(a), P1 and P2 could interfere with the viral infections of *P. aeruginosa* by TFP-specific phages such as PP7 and MPK7, whereas the interaction between PilB and Tip was specifically inhibited by P1, but not by P2 (Figure 3(b)). The inhibitory effect of P2 on viral infections might be due to the viscosity of P2. It should be noted that only P1 was able to inhibit the twitching motility of PA14 (Figure 3(c)), which is a good agreement with the result in Figure 3(b). The twitching inhibitory activity of P1 was evident as well in other twitching-proficient *P. aeruginosa* strains [23] (Figure S1). To further confirm that the observed TFP dysfunction by P1 was the consequence of the PilB delocalization, the polar localization of PilB was examined upon P1 administration into *P. aeruginosa* cells expressing PilB-GFP [21]. As shown in Figure 3(d), the predominant delocalization of PilB was observed in the cells upon P1 treatment, in contrast to treatment of a non-inhibitory peptide, P6, suggesting that the synthetic peptide P1 is sufficient for the Tip function by exogenous administration to *P. aeruginosa*.

(a) Partial sequence alignment between Tip and its homologs of the related phages. A series of 6 peptides (P1 to P6) were designed with the spanned regions designated as colored horizontal lines. Each peptide consisted of 15–16 aa long with a 5-6-aa overlap between the neighboring peptides in a row. The critical regions for the Tip activity are designated in blue colors as in Figure 1(a). (b) Chemical synthesis of the Tip-derived peptides in (a). The sequences of the 6 peptides are shown with C-terminal amidation. The underlined residues (red and green) of P1 denote those subjected to the point mutation analysis (see texts and Figure 5). Asterisk (*) indicates the discarded peptide due to its poor aqueous solubility (P5).

(a) Interference with TFP-specific phage infection. Ten and 100 plaque forming units (pfu) of PP7 and MPK7 phages [23,32] were mixed with the synthetic Tip-derived peptides (5 mM) in Figure 2b, except for P5 with poor solubility. The phage-peptide mixtures were spotted onto the lawn of PAO1. The TFP inhibition activities were assessed by the peptide-mediated reduction of pfu. (b) Interference with PilB-Tip interaction. *E. coli* stain, DHP1 containing pUT18C and pKT25 (vector), pUT18C-Zip and pKT25-Zip (Zip/Zip), and pUT18C–PilB and pKT25-Tip (PilB/Tip) were incubated with either nothing (-) or one of the synthetic peptides (P1, P2, and P6) for 10 min and spotted on a MacConkey agar supplemented with 1% maltose. (c) Twitching inhibition. Five microliters of either TDW (-) or one of the synthetic peptides (5 mM) were directly treated to the PA14 colonies on LB agar for 10 min, and then the colonies were used for twitching motility assay. The twitching zones were stained with 0.1% crystal violet. (d) PilB delocalization. The PA14 cells containing PilB fused with GFP [21] was treated with either TDW (-) or one of the synthetic peptides (1 mM) for 10 min. PilB delocalization was monitored by fluorescence microscope as described in Methods. The polar (i.e. both unipolar and bipolar) localization of PilB in the cells was indicated as a red arrow. (e) Quantitation of PilB delocalization. Each fraction of PilB localization (polar, empty; diffuse, filled) in the independent fields was measured and represented with error bars represent the standard deviations. Statistical significance between the groups is indicated, based on a *p* value of less than 0.001 (***) by Student’s *t* test.

**Figure 4.**
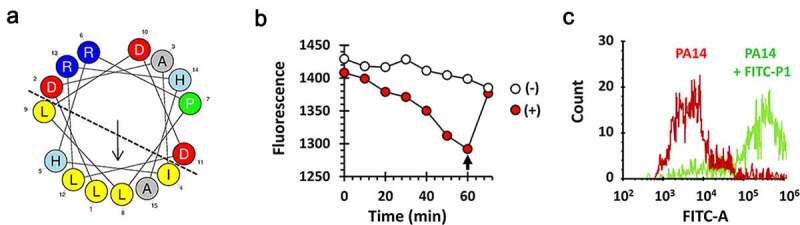
Structure and membrane permeability of P1

P1 is predicted to be a unique amphipathic anionic cell-penetrating peptide comprising α-helical motifs [24], with the hydrophilic residues concentrated on one side of the helix and hydrophobic residues on the other side (Figure 4(a)), and thus was deemed capable of penetration of the bacterial cell wall and membrane to reach its target protein, PilB in the cytoplasm. To assess the bacterial permeability to P1, we conjugated P1 with FITC fluorescein and measured the ability of the FITC-P1 conjugate to interact with or enter into the artificial liposome consisting of phosphatidylethanolamine, phosphatidylglycerol and cardiolipin resembling the *P. aeruginosa* cell membrane [25]. The fluorescence was measured after mixing the FITC-P1 conjugate and the liposome (Figure 4(b)). The fluorescence gradually disappeared over time, suggesting that FITC-P1 could indeed interact with or enter into the liposome that quenches the fluorescence. The liposome-mediated fluorescence quenching was also confirmed by disrupting the liposome with a nonionic detergent (Triton-X100). This was further substantiated by another experiment using flow cytometry (Figure 4(c)). *P. aeruginosa* cells that had been incubated with the FITC-P1 conjugate showed significantly higher intracellular fluorescence intensity. These results suggest that the synthetic peptide P1 that spans from Leu73 to Ala87 might be sufficient for PilB dysfunction upon exogenous administration.

(a) Helical wheel projection of P1. The hydrophobic and hydrophilic residues are separated by a dotted line. The arrows indicate the helical moment. Amino acid color codes: hydrophobic, yellow; cationic, dark blue; anionic, red; alanine and glycine, gray; proline, green; histidine, light blue. (b) Fluorescence quenching of the FITC-P1 conjugates by liposome. The liposome composed of phosphatidylethanolamine, phosphatidylglycerol and cardiolipin was prepared to mimic the *P. aeruginosa* membrane. FITC-P1 (64 μM) was incubated with noting (-; empty) or the liposome (+; filled). Triton-X100 was added to the mixture after 60 min (arrow). Fluorescence from free (i.e. untrapped) FITC-P1 was observed at the designated time points. (c) FACS analysis of the PA14 cells after FITC-P1 treatment. PA14 cells were incubated with noting (red) or FITC-P1 (64 μM) (green) for 30 min, and then washed with PBS. Fluorescence intensity of the cells was analyzed by flow cytometry, as described in Methods.

### P1 contains the critical amino acid residues for the Tip function

**Figure 5.**
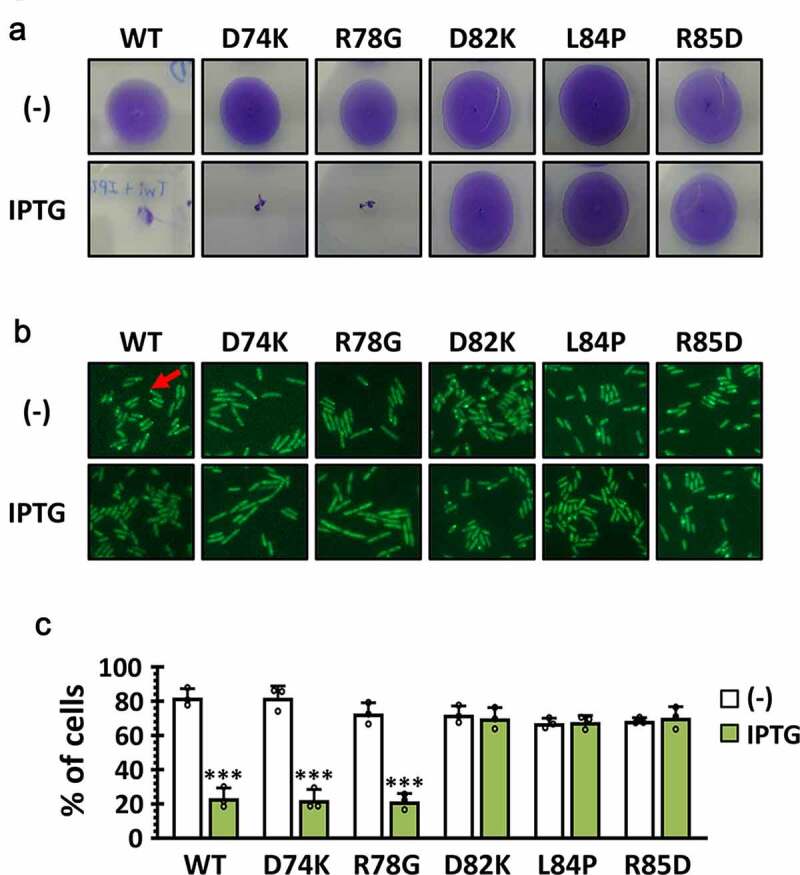
Amino acid residues in P1 critical for the Tip function

It was intriguing that P1 contains the 8 amino acids in the dispensable region, since NΔ80 was fully functional (Figure 1). This has led us to the suggestion that the C-terminal 7 aa of the P1 (i.e. LDDLRHA) might be responsible for the direct interaction with PilB. To corroborate the role of the 7-aa region of Tip in PilB function, we generated 5 point mutants, three of which have the codon changes at the 7-aa region (D82K, L84P, and R85D) and two of which have the codon changes at the other (i.e. 8-aa) region (D74K and R78G). As shown in Figure 5, three mutants (D82K, L84P, and R85D) for the 7-aa region did not inhibit twitching motility and PilB localization at all, whereas the other two mutants phenocopied the wild type in twitching motility and PilB localization. This result substantiated that the 7-aa region of P1 was critical for the Tip function in regard to inhibiting its target, PilB.

Twitching motility (a) and PilB localization (b) were assessed using the PA14 cells harboring the wild type (WT) Tip and its point mutants (D74K, R78G, D82K, L84P, and R85D) for the amino acids within the P1 region. Twitching motility was examined in the absence (-) or presence of 1 mM IPTG as in Figure 1(a) and PilB localization was monitored in the presence of 1 mM IPTG by fluorescence microscope as in Figure 3(d). The polar (i.e. both unipolar and bipolar) localization of PilB in the cells was indicated as a red arrow. (c) Quantitation of PilB localization. The percentage of polar localization of PilB was determined in the absence (empty) or presence (filled) of 1 mM IPTG, with the error bars representing the standard deviations. Statistical significance between the groups is indicated, based on a *p* value of less than 0.001 (***) by Student’s *t* test.

### P1 shows therapeutic efficacy in Drosophila infection model

**Figure 6.**
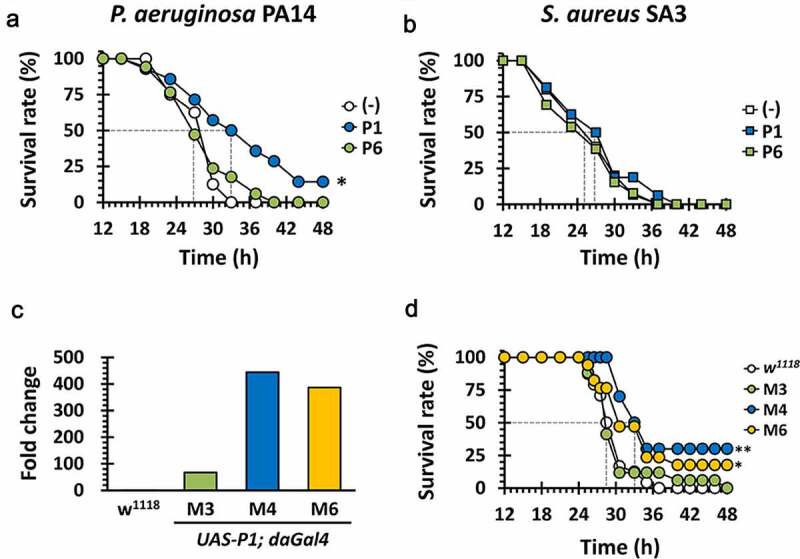
Antibacterial efficacy of P1 in vivo

Experimental verification of the anti-PilB activity of P1 in vitro led us to assess its antipathogenic activity in vivo, in that PilB is required for the full virulence of *P. aeruginosa*. To this end, we have chosen the *Drosophila* systemic infection model, as described for the antibacterial activity of the peptide antibiotic, polymyxin B [26]. Either P1 or P6 was administered to the flies by pricking with bacterial suspensions. As shown in Figure 6(a), P1 displayed antibacterial activity by significantly delaying the *P. aeruginosa* PA14-induced killing of *Drosophila*. In contrast, P6 did not show any antibacterial activity at all. It was intuitively understandable but should be noted that the antibacterial activity of P1 was specific to *P. aeruginosa*, in that flies infected by methicillin-resistant *Staphylococcus aureus* (MRSA) SA3 were not rescued upon P1 administration (Figure 6(b)). Pollitt et al. demonstrated that *S. aureus* lacks the observable surface appendages such as TFP [27]. It should be also noted that the bacterial growth was not significantly affected by treatment of both P1 and P2 peptides (Figure S2). This result confirmed the in vitro anti-PilB activity of P1 could be translated to the target-specific antipathogenic activity to attenuate the virulence of *P. aeruginosa*.

(a) and (b). Mortality of PA14 and SA3-infected flies fed with P1. The wild-type Oregon R flies were infected with PA14 or SA3 cells that had been co-treated with nothing (empty) or 10 mM of the synthetic peptide P1 or P6 (filled). The survival rates were determined over time for the infected flies up to 48 h post-infection. (c) Ectopic P1 expression in transgenic flies. The constitutive transcriptions of P1 in *UAS-P1*/*daGAL4* fly lines (M3, M4 and M6), which are isogenic mutants of *w^1118^*, were measured by RT-RTi PCR using total RNA extract, as describe in Methods. The relative transcriptional fold changes of P1 were determined in comparison to their parental strain *w^1118^* and normalized using the reference gene *rp49*. (d) Mortality of ectopic P1-expressing transgenic flies upon PA14 infection. The *w^1118^* (empty) and its isogenic *UAS-P1*/*daGAL4* fly lines (M3, M4 and M6) (filled), which constitutive overexpresses P1, were systemically infected by PA14 pricking. Those infected flies were transferred to the new corn meal media and the survival rates were measured up to 48 h post-infection. The dotted lines represent the time required to reach 50% mortality. The statistical significance based on a log-rank test is indicated (***, *p*< 0.001).

To further evaluate the therapeutic efficacy of P1 using this genetically tractable animal model, we generated transgenic flies expressing the 15-aa P1 peptide under the control of the GAL4 driver [28]. The crossing scheme from the heterozygote harboring the *UAS-P1* gene integrated into the second chromosome to the homozygotic flies used for the infection experiments was depicted in Figure S3. We have chosen two transgenic lines (M4 and M6) based on the increased transcription of P1, as assessed by quantitative PCR (qPCR) of the P1 transcript (Figure 6(c)). As shown in Figure 6(d), the PA14-induced *Drosophila* killing was significantly attenuated with reduced mortality scores (~75%) in the transgenic flies (M4 and M6), but not at all in the wild-type control. The virulence-attenuation was not observed either in the PA14-infected control transgenic flies (M3), whose P1 expression was significantly lower (Figure 6(c)). These results suggest that the twitching inhibitory peptide P1 specifically attenuate the acute virulence caused by *P. aeruginosa* in the experimental infections.

## Discussion

Recent advances in phage research have geared much attention to phage-based antibacterial platforms not only for the treatment options to cure bacterial infections, but also for the new maneuvers to identify the bacterial targets [6,29,30]. Phages have been exploited *per se* as the antibacterial agents in case of the bacterial infections that are hardly cured by the currently available antibiotic regimens. In addition to this conventional use of phage therapy, phages are considered as the knowledge platforms to identify new bacterial targets, because phages are the evolving being that are able to sagaciously hijack host machinery during the phage lifecycle. This ability that are acquired by evolutionary prey-predator interaction has led us to harness the phage lifecycle for antibacterial development [8]. Liu et al. [31] first recruited a high-throughput approach to identify antibacterial gene products and their bacterial targets, which enabled them to further identify chemical hits that exhibit antibacterial activities by inhibiting those targets. This strategy has been framed as “phage-inspired antibacterial discovery”, which require continued identification of more and more bacterial targets that are involved in the key processes of bacterial physiology and biologically vulnerable to phage-derived proteins during the phage lifecycle [32–34].

Type IV pili (TFPs) are the bacterial dynamic filamentous appendages that have multiple functions: they are required for DNA uptake and phage uptake [35,36]: they are also required for surface attachment, twitching motility and biofilm formation [37–38], and thus considered as an important mechanosensor in *P. aeruginosa* and *Neisseria* species [39,40]. The aforementioned multiple functions of TFPs regarding bacterial pathogenesis render the TFP biogenesis and/or function as an ideal and attractive target for antibacterial discovery. In this regard, we previously identified a phage protein (Tip) and its bacterial target (PilB) in *P. aeruginosa* [21]. Tip is deemed involved in generalized superinfection exclusion, because PilB is the ATPase required for the TFP assembly that act as the primary receptor for many *P. aeruginosa* phages [23,41,42].

To translate this finding to new antipathogenic discovery, we had hypothesized that a chemical mimetic that mimics the Tip in directly binding to PilB would be the ideal anti-PilB antipathogenic hit. However, the current lack of the structural details of the molecular interaction between Tip and PilB as well as the assay system for high-throughput examination of PilB dysfunction has prompted us to utilize the Tip itself as the anti-PilB hit by identifying the minimal scaffold for PilB dysfunction. As the result, we have identified the synthetic 15-aa peptide P1 that spans from Leu73 to Ala87, where the N-terminal 8-aa (i.e. Leu73 to Leu80) is deemed inessential for the Tip function. Based on the involvement of the C-terminal amino acids in the Tip function and the anti-PilB activity of P1 upon exogenous administration, we suggest that the C-terminal region of Tip could contribute the Tip function either by enhancing the protein stability of Tip or by stabilizing the molecular interaction between the P1 region and PilB, where the 7 amino acids (LDDLRHA termed as P1.1) are critical for the direct interaction with PilB. Since we previously identified the 40-aa Tip-binding internal region of PilB [21], the molecular interaction between this peptide and the 40-aa region should be experimentally envisioned based on a virtual computational modeling of the P1.1-PilB interaction at the 40-aa Tip binding region. And, more importantly, further structural and pharmaceutical optimization to improve the antibacterial efficacy and the druggability can be performed on the 7-aa peptide scaffold by mutations and/or fusion with other functional modalities.

The TFP machinery is ubiquitously found in many Gram-negative bacteria, with the PilB-like ATPases highly conserved in Type II secretion system (T2SS) as well [43]. Furthermore, the 40-aa Tip-binding region was found with 100% sequence identity in various Gammaproteobacterial proteins, as exemplified in the secretory protein, GspE of *Enterobacter cloacae*. Therefore, we expect that this anti-PilB scaffold would be further explored as a new antipathogenic hit that inhibits group motility and toxin secretion in bacterial pathogens. Based on our previous observation that the mutations in the Tip-binding region of PilB resulted in the loss of twitching motility and virulence, the emergence of resistance mutations in PilB might be likely mitigated even upon this antibacterial treatment. In conclusion, our finding suggests that more works need to be done for phage-inspired antipathogenic discovery based on the yet-to-be discovered phage proteins to target the virulence not the growth of the host bacterial pathogens.

## Materials and methods

### Bacterial strains and culture conditions

The bacterial strains used in this study are listed in Table S1. *P. aeruginosa* and *E. coli* strains were grown at 37°C using Luria-Bertani (LB) (1% tryptone, 0.5% yeast extract and 1% NaCl) broth or on 2% Bacto-agar solidified LB plates. Overnight-grown cultures were used as inoculum (1.6 × 10^7^ cfu/ml) into fresh medium and grown at 37°C until logarithmic (OD_600_ of 0.7) phase, and then the cell cultures were used for the experiments described herein. Antibiotics were used at the following concentrations (μg/mL): carbenicillin (200), and Gentamicin (50) for *P. aeruginosa*; ampicillin (50), gentamicin (25) and kanamycin (25) for *E. coli*.

For growth curve determination, *P. aeruginosa* PA14 cells were used as inoculum (2.0 × 10^8^ cfu/ml) into LB containing antibiotics and peptides (if required) and the the cultures were incubated in the microplate reader (EPOCH2, USA) at 37°C for 18 h. During the incubation, OD_600_ was measured at every 30 min.

### Construction of truncated mutants

The truncated mutant constructs for Tip were generated by 4-primer SOEing (splicing by overlap extension) PCR and the resulting PCR products were cloned into pUC18T-mini-Tn*7*-LAC (mTn7lac) for IPTG-inducible expression or pKT25 for bacterial two hybrid assay as described elsewhere [21]. These plasmids were introduced into *P. aeruginosa* PA14 and the *E. coli* DHP1 by conjugation and electroporation, respectively.

### Bacterial two-hybrid assay

A bacterial adenylate cyclase two-hybrid system for Tip-PilB interaction was performed as described previously [44]. The bait and the prey vectors are pKT25 (for the Tip derivatives) and pUT18C (for PilB), respectively. The recombinant pKT25 derivatives were cotransformed with pUT18C-PilB into the *E. coli* DHP1 reporter strain by electroporation. Electroporation was performed at a capacitance of 25 μF, a resistance of 200 Ω, and a voltage of 2.5 kV using a Gene Pulser (Bio-Rad, USA). The transformants were grown on LB agar containing 40 μg/mL X-Gal for 2 d at 30°C or MacConkey agar containing 1% maltose.

### Twitching motility assay

A single colony from the overnight cultures on an LB agar plate was picked with a toothpick and stab-inoculated through a 3-mm thick of 1.5% LB agar plate to the bottom of the Petri dish. After incubation for 48 h at 30°C, a bacterial twitching zone at the interface between the agar and the surface was visualized by 0.1% crystal violet staining.

### Synthesis of peptides

The peptides were designed, synthesized, and C-terminally amidated by standard solid-phase methods using 9-fluorenylmethoxycarbonyl chemistry and then purified by HPLC. The purified peptides were confirmed by matrix-assisted laser desorption ionization mass spectrometry (MALDI II; Kratos Analytical, Japan) and dissolved in water prior to use.

### Phage infection competition assay

The phage strains (PP7 and MPK7) were enriched by a plate lysate method and then the plaque forming units (pfu) of the phage lysates were determined as described previously [23]. The phage aliquots containing 10 or 100 pfu were mixed with the synthetic peptides (5 mM). The phage-peptide mixtures were spotted onto lawn of PAO1. After 24-h incubation, the plaques with clear zone of lysis were examined.

### PilB delocalization assay

The GFP-fused *pilB* gene cloned into pUCP18 (pUCP18-gfp-pilB) was introduced into the PA14 cells that possess mTn7lac-based integration for IPTG-inducible expression of Tip and its derivatives. The bacterial cells were grown on LB agar plates in the absence or presence of 1 mM IPTG. The PilB localization was monitored as the fluorescence visualized using an Axio 5 fluorescence microscope (Carl Zeiss, Germany) at 488-nm excitation.

### Structure modeling and membrane association assay

For structural modeling of P1, the helical wheel projection was generated using HeliQuest (http://heliquest.ipmc.cnrs.fr/cgi-bin/ComputParams.py). FITC-conjugated P1 (FITC-P1) was prepared for cell penetration assays by conjugating FITC and P1. Briefly, the mixture of was incubated for 4 h at room temperature in the dark, and then reacted with 1 M of ethanolamide for additional 2 h. After conjugation, the final product was purified by sephadex G-50 gel filtration and analyzed by reverse-phase HPLC. For fluorescence quenching assay by liposome, the bacterial membrane-mimicking liposome was prepared by gently mixing phosphatidylethanolamine, phosphatidylglycerol, and cardiolipin with 6:2:1 (v:v:v) ratio. The reconstituted liposomes were incubated with the FITC-P1 (64 μM) for 70 min. At 60 min, the liposome leakage was induced by triton X-100. The free FITC-P1 was measured by its fluorescence intensity at the designated time points. For fluorescence-activated cell sorting (FACS) analysis, the 100 µL of PA14 cell was treated with noting or FITC-P1 (64 μM) for 30 min. The treated cells were harvested and washed twice with PBS (137 mM NaCl, 2.7 mM KCl, 10 mM Na_2_HPO_4_ and 1.8 mM KH_2_PO_4_) to remove the free FITC-P1. Fluorescence intensity of the cells were analyzed using a CytoFLEX flow cytometer (Beckman Coulter, USA).

### Construction of transgenic fly lines

For pUAS-P1 expression transgenic fly construction, the P1 region of Tip was codon-optimized for *Drosophila melanogaster*, synthesized, and then cloned into a drosophila gene expression vector, pUAST [45]. The resulting plasmid (pUAST-P1) was used for P-element injection to *w^1118^* (Bestgene Inc. USA), which generated transgenic lines (*UAS-P1*/*CyO*). The transgenic fly lines were then appropriately crossed with a GAL4 driver strains to obtain the balanced heterozygotes (*UAS-P1*/*CyO-GFP; daGAL4*/*MKRS*) as schematically depicted in Figure S1. These heterozygotes were maintained and used to obtain the flies homozygous for *UAS-P1* and *daGAL4*, which were used for experiments.

### RNA assay for P1 expression

RNA extraction was performed from transgenic male flies using TRIzol® (Invitrogen, USA). The RNA samples were subjected to RNeasy Cleanup kit (Qiagen, USA) to completely remove residual DNA. For quantitative measurement of the P1 RNA levels in the transgenic flies, quantitative PCR (qPCR) followed by cDNA synthesis was done by using 1 μg of the RNA samples, ReverTra Ace™ qPCR RT Kit (Toyobo, Japan) and TUNDERBIRD™ SYBR® qPCR Mix (Toyobo, Japan) as described previously [46]. The primers used for RNA assay are listed in Table S2, with the *rp49* gene as the control.

### Measurement of antibacterial efficacy

*Drosophila* systemic infection was performed as previously described [47]. Briefly, *D. melanogaster* strain Oregon R, *w^1118^* and its fly lines were grown and maintained at 25°C with 50% humidity in a 12 h light/12 h dark cycle using the corn meal-dextrose medium [0.93% agar, 6.24% dry yeast, 4.08% corn meal, 8.62% dextrose, 0.1% methyl paraben, and 0.45% (v/v) propionic acid]. For systemic infection, 4- to 5-day-old adult female flies were infected by pricking at the dorsal thorax with a 0.4 mm needle (Ernest F. Fullam, Inc.). The needle was dipped into PBS-diluted bacterial suspension containing either *P. aeruginosa* PA14 (10^7^ cfu/ml) or *S. aureus* SA3 [48] (10^7^ cfu/ml) grown to the OD_600_ of 3.0. The bacterial suspensions were injected with either P1 or P6 (10 mM). Survival rates of the infected flies were monitored for up to 72 h post-infection. Flies that died within 12 h for PA14 and SA3 were excluded in mortality determination. Mortality assay was repeated at least four times.

### Statistics

Statistical analysis was performed using GraphPad Prism version 8.0 (GraphPad Software, USA). Data for each analysis represents a least set of 3 independent replicates. Statistical significance between the groups is indicated, based on a *p* value of less than 0.001 (***, *p* < 0.001) by using Kaplan-Meier log-rank test and Student’s *t* test. Error bars represent the standard deviations.

## Supplementary Material

Supplemental Material
