# Supplementary figures and images for: A phage protein-derived antipathogenic peptide that targets type IV pilus assembly
Source: Virulence. 2021 May 19;12(1):1377–87. doi: 10.1080/21505594.2021.1926411 (PMC8143254; doi:10.1080/21505594.2021.1926411)

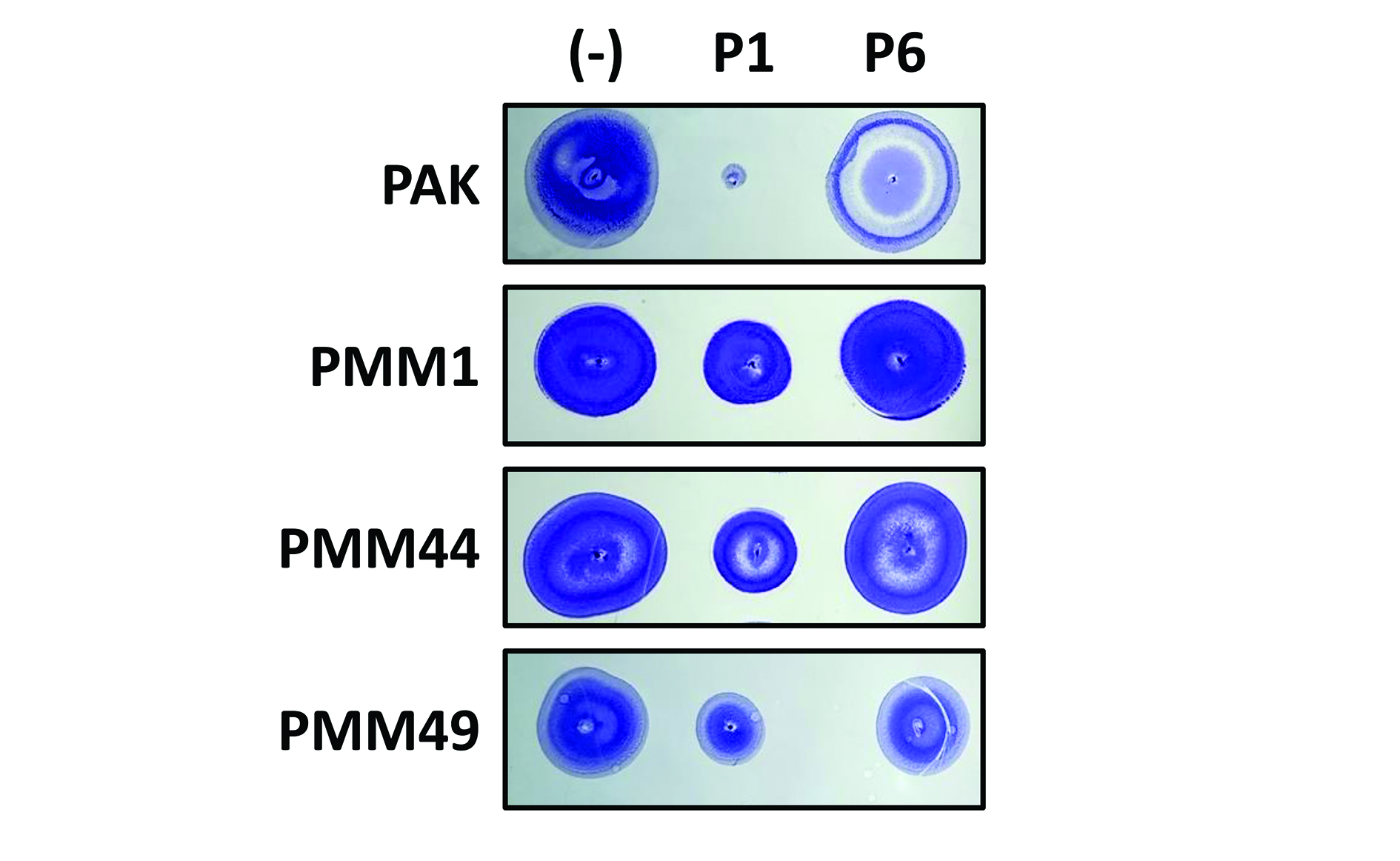

Supplement: Supplemental Material [file KVIR_A_1926411_SM3235.zip › 1926411(supplementary)/ChungIY_P1_FigS1.tif]

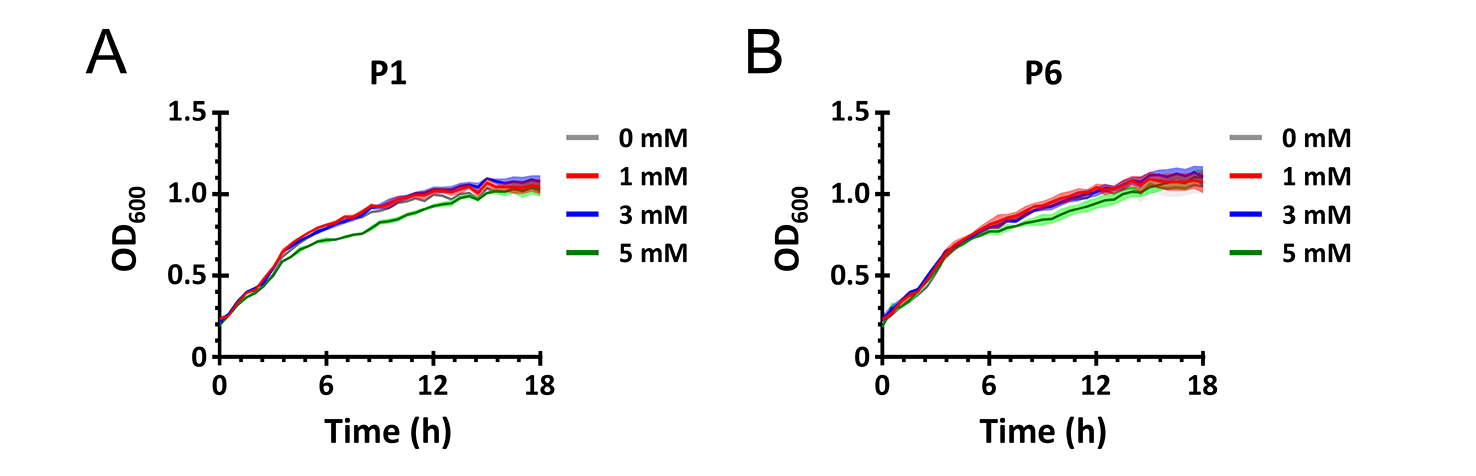

Supplement: Supplemental Material [file KVIR_A_1926411_SM3235.zip › 1926411(supplementary)/ChungIY_P1_FigS2.tif]

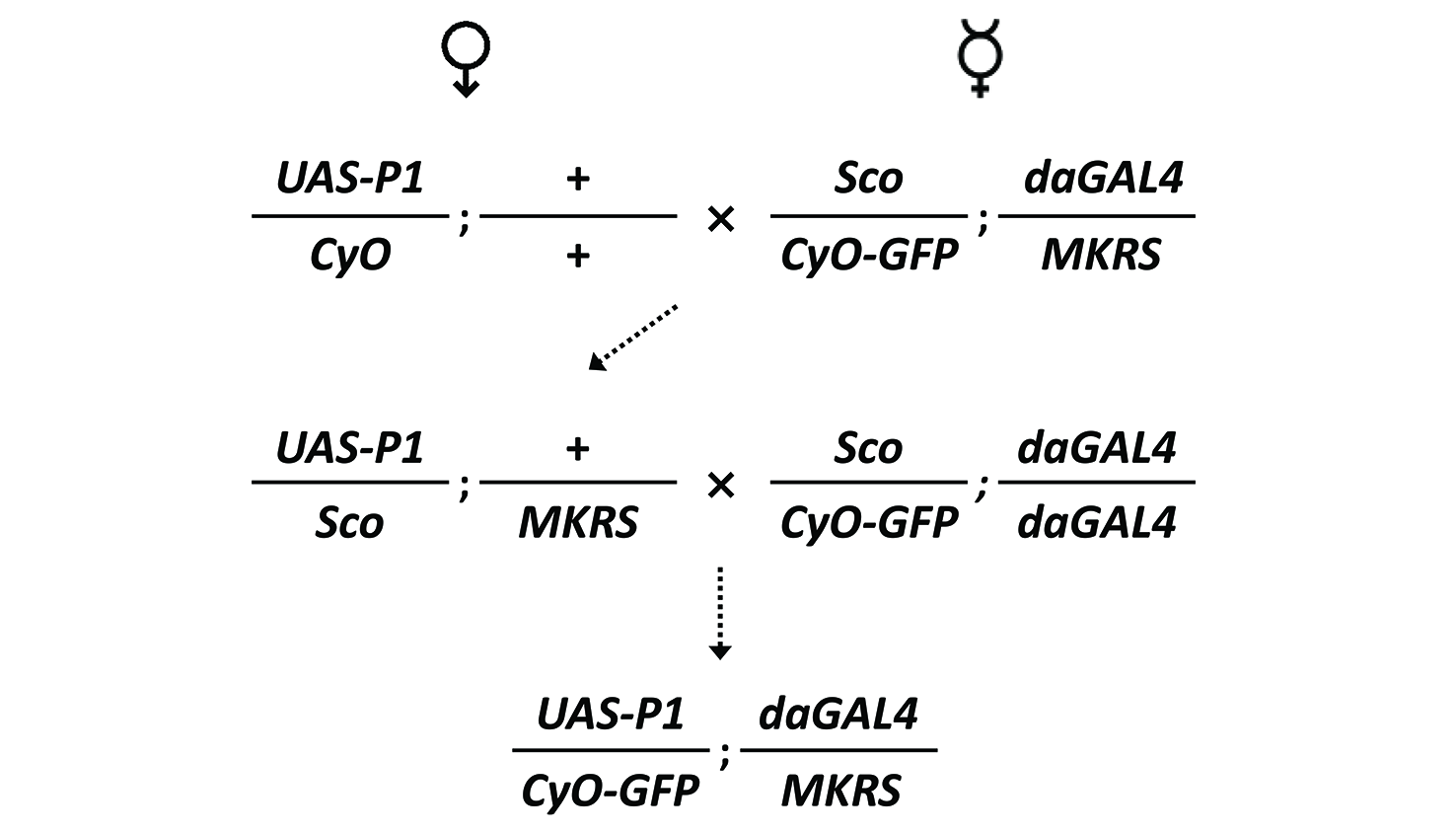

Supplement: Supplemental Material [file KVIR_A_1926411_SM3235.zip › 1926411(supplementary)/ChungIY_P1_FigS3.tif]
